# Does the *Janani Suraksha Yojana* cash transfer programme to promote facility births in India ensure skilled birth attendance? A qualitative study of intrapartum care in Madhya Pradesh

**DOI:** 10.3402/gha.v8.27427

**Published:** 2015-07-07

**Authors:** Sarika Chaturvedi, Ayesha De Costa, Joanna Raven

**Affiliations:** 1 Department of Public Health and Environment, R D Gardi Medical College, Ujjain, India; 2 Department of Public Health Sciences, Karolinska Institutet, Stockholm, Sweden; 3 Department of International Public Health, Liverpool School of Tropical Medicine, Liverpool, UK

**Keywords:** observations, midwifery, JSY, India, skilled birth attendance, quality of care, hospital birth, cash transfers, qualitative research

## Abstract

**Background:**

Access to facility delivery in India has significantly increased with the Janani Suraksha Yojana (JSY) cash transfer programme to promote facility births. However, a decline in maternal mortality has only followed secular trends as seen from the beginning of the decade well before the programme began. We, therefore, examined the quality of intrapartum care provided in facilities under the JSY programme to study whether it ensures skilled attendance at birth.

**Design:**

1) Non-participant observations (*n*=18) of intrapartum care during vaginal deliveries at a representative sample of 11 facilities in Madhya Pradesh to document what happens during intrapartum care. 2) Interviews (*n*=10) with providers to explore reasons for this care. Thematic framework analysis was used.

**Results:**

Three themes emerged from the data: 1) delivery environment is chaotic: delivery rooms were not conducive to safe, women-friendly care provision, and coordination between providers was poor. 2) Staff do not provide skilled care routinely: this emerged from observations that monitoring was limited to assessment of cervical dilatation, lack of readiness to provide key elements of care, and the execution of harmful/unnecessary practices coupled with poor techniques. 3) Dominant staff, passive recipients: staff sometimes threatened, abused, or ignored women during delivery; women were passive and accepted dominance and disrespect. Attendants served as ‘go-betweens’ patients and providers. The interviews with providers revealed their awareness of the compromised quality of care, but they were constrained by structural problems. Positive practices were also observed, including companionship during childbirth and women mobilising in the early stages of labour.

**Conclusions:**

Our observational study did not suggest an adequate level of skilled birth attendance (SBA). The findings reveal insufficiencies in the health system and organisational structures to provide an ‘enabling environment’ for SBA. We highlight the need to ensure quality obstetric care prior to increasing coverage of facility births if cash transfer programmes like the JSY are to improve health outcomes.

Increasing the proportion of women who deliver in health facilities has been a primary strategy of many governments and donors to reduce maternal mortality. The Indian government has employed this strategy where programmes focussed on encouraging all women to deliver in a facility have been implemented. When the majority of women access health facilities for delivery care, outcomes are largely dependent on quality of care at health facilities (1). Good quality care is essential not only in emergencies but also in routine care for normal deliveries to ensure timely detection of complications that can prevent adverse outcomes and promote future use of delivery care services.

In India, the rise in coverage of institutional care for delivery has been steep from 41% in 2004 to 73% in 2012 (2). This has largely been achieved through the implementation of a nationwide conditional cash transfer programme – Janani Suraksha Yojana (JSY), meaning safe motherhood scheme (3) launched in all states of India in 2005. The JSY pays cash to women on delivering at health facilities, the incentive varying from about US$22 in poorer states where all women are eligible for it to about US$11 in others where only the poor are eligible. The JSY aims at reducing maternal and neonatal mortality by promoting facility births. The JSY was launched as a flagship scheme of the health system reform in India – the National Rural Health Mission – that committed to increased public spending on health and ‘architectural corrections’ in the health system such as decentralisation of funds, introduction of district-level management units and information systems, improved authority to local institutions, and increased number of staff; at the same time as the introduction of the JSY, measures to improve staff skills at maternity care through the 3 weeks Skilled Birth Attendant training were implemented. Despite the steep increase in facility births, maternal mortality decline follows a similar trend to before the introduction of the JSY. Studies thus far have been unable to detect an effect of the steep rise in facility births during the JSY on maternal mortality reduction (4). A recent study finds no association between increased facility births and reduction in maternal mortality in India and suggests poor quality of care at facilities to be a potential reason for this (5). The purpose of promoting facility births is to ensure skilled attendance at birth that implies care is provided by competent personnel in settings adequately equipped to provide appropriate care for normal deliveries and manage complications (6). Facility births can be expected to improve maternal health outcomes, only if in-facility care is of an acceptable standard and ensures skilled attendance at birth. Hence, an important question is whether increased access to institutional birth under the JSY programme ensures skilled attendance at birth for programme beneficiaries.

To answer this question, we conducted a detailed exploration of the quality of intrapartum care provided to women in facilities under the JSY programme using a qualitative paradigm. We report findings from non-participant observations of intrapartum care at different levels of facilities under the JSY in Madhya Pradesh and subsequent interviews with providers to explore reasons for the practices we observed. We discuss the implications of these findings for the national and global maternal health agenda.

## Methods

### Study setting

This study was conducted in Madhya Pradesh (MP) province in central India. This is a large province with a population of 72 million, with over one-third living in rural areas and agriculture is the main source of income. MP has one-third of its population living below the official poverty line and has amongst the poorest health indicators in the country. The private health sector is small, concentrated in urban areas, and unaffordable for the majority. The JSY programme in MP has functioned largely through public sector facilities, all of which implement the programme. Being a poor state, all women in MP are eligible to participate in the JSY. Since the implementation of the JSY, institutional delivery proportion in MP increased from 31% in 2007 to 72% in 2012 (2).

The health system in MP has a three-tiered structure as in the rest of the country. At the lowest tier is a Primary Health Centre (PHC); while at the second tier is a Community Health Centre (CHC); and the district hospitals (DHs) form the third tier. The PHCs have 4–6 beds and provide care for normal delivery and referral in case of complications. The CHCs are 30-bedded secondary care centres with specialist services and some are designated as First Referral Units, while the DHs are the apex institutes in the district that provide caesarean section and care for women with complications, as well as routine care. Although distribution might vary depending on district size, each district has approximately 30 PHCs, 5–7 CHCs, and 1 DH. The facilities in MP are understaffed with large vacancies of nursing cadres (33%) (7). Delivery service providers at PHCs are generally auxiliary nurse midwives (ANMs), who undergo an 18-month pre-service training, while at CHCs and DHs, they are mostly staff nurses with a 4-year bachelor’s degree in nursing or those who undergo the 3.5-year training to be general nurses and midwives (GNMs). There is no separate cadre as ‘midwife’ but they undergo a combined nursing and midwifery training. ANMs are referred to as ANM, while GNMs or BSc. nurses are called staff nurses. Seniority is by years of service, generally the most senior nurse serves as the nurse-in-charge or head nurse. Doctors and specialists if available in the CHCs and DHs attend to delivery patients if called for by nurse-midwives. Other than qualified staff, the delivery rooms have untrained female support staff, that is, the ‘sweeper’, who is responsible for cleaning the delivery room and the ‘ayah’ who is expected to assist the nurse with postnatal and newborn care such as supporting breastfeeding, weighing, and bathing the baby. The ayah learns her role and skills ‘on the job’. Along with the JSY, the government also created a cadre of female village-level health volunteers called ASHAs (accredited social health activists) who escort women to institutions for delivery. It is customary that female family members accompany women to the facility and through the delivery; these attendants provide emotional support to the labouring woman.

### Study districts and facilities

We collected data from three districts in MP. Districts are administrative units within a province, each with a population of 1–1.5 million. Of the 50 districts in MP, three heterogeneous districts were selected for this study based on their varying geographic location and differing human development indices, proportion of institutional births, and maternal mortality ratio (MMR) that represent different levels of socio-economic development, programme uptake, and maternal health outcomes. (Table 1 shows the latest figures at the time of district selection.)

**Table 1:** Study districts

Indicator	District 1	District 2	District 3	Madhya Pradesh (range for districts)
Institutional births (%)^a^	59	83	75	79 (48–93)
Maternal mortality ratio^a^	415	206	386	277 (202–415)
Human Development Index^b^	0.4	0.6	0.5	(0.3–0.7)

a *Source*: Annual Health Survey 2011–2012, Government of India

b Human Development Indices of districts of MP, Human Development Report 2007.

We selected study facilities to include the range of facility levels in the public health system covered by JSY. As the JSY programme was rolled out in all public facilities in MP and nationally at the same time, it was not possible to study care in facilities without the programme.

In each district, we selected a DH and two CHCs that performed the highest and second highest number of deliveries in the last year as per district records. We chose to select such functioning facilities to make it feasible to conduct the observations and to present how care is provided in the JSY programme facilities generally. In districts 1 and 2, we also included the PHC that performed the highest number of deliveries in the last year. The study included 11 facilities in the JSY programme and are the dominant sources of obstetric care in the districts. The number of deliveries in the month prior to this study ranged from 214 to 890 at the selected DHs, from 143 to 290 at the selected CHCs, and from 52 to 90 at the selected PHCs.

### Observation of deliveries

The observers visited study facilities along with colleagues undertaking a survey of facilities as a part of a larger project (MATIND). The timing of these facility visits was unannounced and each visit lasted five consecutive days, which allowed for all data collection activities including observation. On reaching each facility, the study team (consisting of five or six members) approached the facility in-charge, explained the purpose of the project, and provided a briefing about the intended data collection methods including observations. The team obtained permission from the in-charge who then took the team on a facility tour, introduced them to the individual staff members, and the team discussed the data collection, to which the staff agreed to participate. Staff were thus accustomed to the observers during the 5-day period and interacted with them easily.

The observers stayed in the maternity unit at the facility and identified women coming for delivery (intrapartum) care. They approached the woman (any woman arriving to seek delivery care) and her attendants (female relatives who accompany women) on their arrival to the facility and explained the purpose of the observations. Informed verbal consent was obtained from the labouring woman and her attendants before the observation. Eighteen deliveries were observed in 11 facilities in the three study districts (Table 2).

**Table 2:** Number of observations of normal vaginal deliveries – distributed by study districts and facility levels

	DH	CHC	PHC	Total
District 1	3	4	1	8
District 2	1	4	1	6
District 3	2	2	0	4
Total	6	10	2	18

Number of deliveries in last month ranged from 214 to 890 in DH, 143 to 290 in CHC, and 52 to 90 in PHC. DH: District Hospital; CHC: Community Health Centre; PHC: Primary Health Centre.

The first author (SC), a trained medical doctor, conducted the observations between February and November 2012. A trained nurse (JK) accompanied SC during the 14 observations that were conducted in districts 1 and 2. The semi-structured nature of the observation guide allowed the observer to ‘look for’ pre-defined elements of care as well as other events or behaviour that may arise during the observation. The guide was informed by the WHO recommendations on care for normal delivery (8), guidelines for skilled birth attendants in India (9), and the authors’ clinical experience of working in public health facilities in India. The observations were non-participant (the observer did not take part in any of the activities), and just observed, except in one life-threatening situation, when the observer conducted newborn resuscitation to save a newborn as the staff expressed inability to resuscitate. Observers collected data on both technical as well as behavioural aspects of care. These elements of data collection were discussed amongst the authors and finalised after pilot testing. SC trained herself in conducting systematic observations during the pilot study using guidance provided by Patton (10). We included observations of normal/vaginal deliveries keeping our objective to study routine delivery practices. Normal delivery services are provided at all levels of health facilities selected for this study, that is, DH, CHC, and PHC. Observations began during the active stage of labour and continued until 1 h postpartum. All processes of care provided during this time and interactions with staff were witnessed. Immediately after each observation, SC made notes which she then expanded within 2 days to produce detailed transcripts. JK reviewed these transcripts, and SC made any necessary changes. As SC became more experienced with conducting the observations, the team felt there was no need to involve JK during observations in the third district. The four observations in district 3 were, therefore, conducted by SC alone using the same method as described above. The team reached data saturation point by the time observations were being conducted in district 3, hence, we conducted fewer observations in this district and did not include the PHC.

### Interviews with staff members

To explore the reasons for the practices being carried out during our observation study, semi-structured interviews were conducted with qualified clinical staff members involved in delivery care in the study facilities. The interviews aimed at gathering opinions on practices in the JSY programme observed across the range of study facilities, hence the interview respondents were not necessarily the ones whom we had observed providing care, but represented them well. Cadre and level of facility were considered to ensure maximum variation in sampling. Staff nurses (GNMs) (6), obstetricians (2), and senior nurses (BSc and GNM) (2) who were responsible for the administration and management of the delivery room in a variety of facilities were interviewed as they would provide rich data on the practices of staff within their facility. The topic guide for the interviews was based on the key issues arising from the observations while it also allowed for probing and exploring any issues emerging during the interviews. The researcher re-visited the facilities where observations took place between September 2012 and October 2013 to conduct the interviews and so there was a time lag of between 2 days and 6 months between the observations and interviews. There were no contextual changes, such as new healthcare policies or projects, or changes in workforce, in the districts during this time. SC conducted all interviews, some entirely in the local language while others were partly in English, at study facilities during working hours. Consent was obtained from each participant prior to starting and recording the interview. Data were transcribed verbatim and translated into English. All transcriptions and translations were checked for accuracy.

#### Ethics statement

The study protocol was approved by the Institutional Ethics Committee at R D Gardi Medical College, Ujjain, India.

#### Analysis

Data from the observations and the interviews were analysed using the thematic framework analysis, which facilitates rigorous and transparent analysis (11). All co-authors were involved in the analysis processes. First, the observation data were analysed. The observation transcripts were read to identify emerging concepts; a coding framework was developed based on these emerging concepts. All transcripts were then indexed using this coding framework. Indexed data were entered into matrices developed for each code, allowing for comparison within and across cases. Maps were then developed to help illustrate the patterns in the data. These matrices and maps were used to describe similar and divergent processes and practices and, develop explanations, and find associations between them. A similar approach was followed for the analysis of the interviews. Analysis of the observation data and the identification of the processes and practices of care informed the development of the coding framework for analysis of interview data.

## Results

The length of the observations ranged from 2 to 8 h, depending on how soon after arrival the woman delivered, and included before delivery, during delivery, and for 1 h after delivery. On arrival, women were examined in the delivery room and depending on their progress in labour, they were asked to wait in the corridor or on the ward, or stayed on the delivery table. After delivery of the placenta, women were sent to the ward. Most women observed in this study arrived close to delivery.

Three themes emerged from the analysis of the observation and interview data. The first theme ‘delivery environment is chaotic’ encompasses the organisational aspects of care. The second theme ‘staff do not provide skilled care’ describes the technical aspects of the care that was observed, while the third theme ‘dominant providers, passive recipients’ describes the behavioural aspects (Table 3). During data analysis, we looked for patterns within the data by level of facility. However, we found little variation in practices across the levels of facilities.

**Table 3:** Thematic description of intrapartum care in facilities under Janani Suraksha Yojana programme in Madhya Pradesh, India

Theme	1. Delivery environment is chaotic	2. Staff do not provide skilled care	3. Dominant staff, passive recipients
Sub themes	i) Delivery rooms are not conducive to safe women friendly care	i) Limited monitoring during labour and immediately after delivery	i) Staff provide some support during labour
	ii) Poor coordination between providers	ii) Some good practices followed	ii) Staff abuse and ignore women during delivery
		iii) Harmful and unnecessary practices coupled with poor techniques	iii) Women are passive and accept dominance/disrespect
		iv) Lack of readiness to provide routine care	iv) Attendants as ‘go-between’ patients and providers

### Delivery environment is chaotic

#### Delivery rooms are not conducive to safe women friendly care

The observations found that the delivery rooms were generally poorly maintained with regard to readiness to provide good quality delivery care. This was observed in several areas: staffing, infrastructure, equipment and supplies, and cleanliness.

*Staffing*: At most CHCs, a single nurse was responsible for the delivery room as well as postnatal mothers in a ward. Sometimes care for inpatients in other wards was also the duty of the delivery room nurse. At DHs, although there were more staff available on duty, only one or two nurses actually conducted the deliveries while others were seen to be occupied with paper work. Hence, the issue is not only of staff shortage but also of effective work distribution amongst the available staff. The work distribution appeared to be *ad hoc* rather than organised in view of patient load and priority to providing prompt services. During the interviews, staff shortage was frequently mentioned as well as the increased workload following the introduction of the JSY.

*Infrastructure*: The general impression of a delivery room was an unclean area with blood spills, cries, and chaos rather than a comfortable, calm, and clean place for delivery as is evident from the following notes:

There were about eight women, seven nurses and one resident doctor in the delivery room. It was chaotic. Curtains between tables were rolled up. People moving in and out of the delivery room, talking loudly, some women crying in pain …. (DH)

Delivery room had doors–both were glass doors and kept open always, there were no curtains; the platform (stone surface on which equipment is placed), sink and movements of staff in the delivery room could be seen from the corridor outside. (CHC)

The delivery rooms were not exclusively used for deliveries. At larger facilities, female outpatients and inpatients were also examined here, often when women were delivering on the adjacent table.

The participant was on the delivery table, bearing down as she had contractions. The delivery room was crowded again; the doctor had just examined about 12 women on the other delivery table, the 12 women and their attendants were standing by with bottles of IV (intravenous) fluid – the nurse was starting an IV drip for each woman turn-by-turn with help from the ayah and the sweeper. Participant asked the mother to cover her with the sari. This crowding went on for about half an hour, thereafter the participant was alone in the delivery room. (CHC)

With the sub-optimal set-up of the delivery room, the staff spent time making alternate arrangements. For instance, placing buckets of water at the sink where tap water was not available.

During interviews, staff also mentioned issues with management of infrastructure such as power shortages and unavailability of power back up in delivery rooms. This was said to affect the use of sterilisers and infant warmers and at times compelled staff to conduct deliveries in candlelight.

*Equipment and supplies*: In all facilities, equipment was generally not well kept and ready to use indicating poor systems of routine maintenance. This was observed to lead not only to chaos and delays but also to life-threatening situations. For instance, when a newborn required resuscitation at a CHC, the neonatal mask was covered with cobwebs, and the infant warmer had no bulbs. There was a power failure at the same time, so to perform resuscitation the newborn was moved to the operating theatre where there was power back up.

Staff reported that they were unable to sterilise instruments in the ideal way. They said that it was routine practice to reuse instruments after washing with tap water or dipping in chlorhexidine solution. In some facilities, the instruments were sterilised once a day. Similarly, surgical gloves and also single use gloves were said to be reused without proper disinfection. Washing and sterilising instruments was the responsibility of the ayahs or sweepers, who, the staff mentioned, do not have adequate knowledge nor understand the importance of infection control.

*Cleanliness*: Staff reported problems with routine maintenance and cleaning of the delivery room. A soap solution was generally used for cleaning the delivery table once a day. The rubber sheet on the table, where available, was wiped clean with a wet cloth, and reused after a delivery, as there were not enough sheets to change after each delivery. They reported that they previously used hypochlorite solution for disinfection, but this was no longer practiced because of the increased numbers of deliveries following the introduction of JSY and the bleach powder not being available.

The staff identified several underlying causes for these problems with quality of care. Irregular and inadequate drug supplies forced staff to prescribe drugs and supplies, which attendants bought from outside pharmacies. Functional basic instruments dedicated for the delivery room were not available. Many issues with staffing were reported. There is an overall shortage of staff, which has been exacerbated by the increase in numbers of deliveries in facilities, created by the JSY. Staff perceived that the government had attempted to overcome the shortage of trained nurses by posting ANMs and para-medical workers in PHCs and CHCs but as they are trained for community work rather than facility care, the quality of care at facilities is compromised. In addition, cleaning staff are now appointed at facilities on short-term contracts. Staff explained that it is difficult to get them to do the work well, as they are paid such low salaries.

#### Poor coordination between providers

Several issues with communication and coordination between providers were observed in the facilities. Clinical examinations findings were sometimes briefly mentioned at staff handover, but a formal and comprehensive communication expected at handover was never seen. At DHs, the staff did not seem to have an opportunity for this in the midst of a succession of deliveries but even when there were opportunities, they were not utilised. Moreover, this exchange of information was dependant on oral communication, as clinical records were never written concurrently. It was often the attendants who informed the next staff about the previous examinations conducted. The following extract from an observation at a CHC describes the lack of communication and coordination and how it may lead to complications.

2 pm. The nurse on morning duty noticed meconium stained liquor and called the doctor to examine the participant. The doctor (paediatrician) examined her and told her the foetal heart rate was about 160 b/min. He then asked the nurse to inform the surgeon for CS. The doctor had neither asked about the drugs given to the participant nor did the nurse mention to him.

2.15 pm. When duty shifts changed, the nurse told the ANM ‘this one (pointing to participant) has thick liquor, inform the doctor (paediatrician who had examined her) about her by 3.30–4 pm’. The nurse also reported that she had given two injections of Epidosin and the IV fluid with sodium bicarbonate infusion and she had prescribed two more injections of Epidosin.

4 pm. The participant had a vaginal delivery; the doctor was called for neonatal resuscitation that he succeeded at.

4.30 pm. Doctor to ANM: ‘Didn’t she (nurse) tell you I had advised Caesarean?’

ANM: ‘She told me to inform you if this woman did not deliver by 4 pm. She delivered about 4, so I did not inform you Sir’.

Doctor: ‘I had told her to inform Sir (surgeon) about taking her for Caesarean’.

The ANM kept quiet.

The long hours spent at the delivery rooms during this study did not give a sense of good teamwork. Strong hierarchical relationships were apparent, with little evidence of professional support and collaboration. For example, in a DH, a patient had postpartum haemorrhage; the obstetrician on duty examined her and ordered 4 ampoules of injection Oxytocin to be given IV. The nurse, however, infused only 2 ampoules and when another obstetrician arrived she asked her to re-examine the patient to confirm that only 2 ampoules of Oxytocin were needed, despite the first doctor advising 4. The nurse seemed more comfortable with the second obstetrician who was older, more senior, and less strict than the first obstetrician.

During interviews, nurses reported that they needed support from peers, but this was rarely provided. Staff nurses were posted with ANMs, and they felt that they were unable to support the nurses as they had inadequate training and knowledge. In all CHCs, nurses were generally left to manage the delivery rooms, with little support from doctors.

### Staff do not provide skilled care

#### Limited monitoring during labour and immediately after delivery

In all study facilities, it was common to see a vaginal examination done at least one or more times for monitoring the progress of the labour. However, these examinations only measured cervical dilatation and neither effacement nor station was judged. Other essential examinations such as blood pressure, pulse rate, temperature, and pallor were rarely performed. The foetal heart was auscultated only in two cases that a doctor examined. Moreover, in both instances the doctor only auscultated for the presence of the foetal heart sounds (FHS) but did not count the rate. The frequency and duration of contractions were not assessed during any of the observations even when mothers were present in the facility several hours before delivery occurred. The findings of the examinations were not documented in the case notes or the partograph, at the time of examination or later during labour or after delivery.

The ASHA or attendants appeared to decide when to take the labouring women to the delivery room based on their judgements of increased severity of contractions. Owing to an absence of professional monitoring of progress of labour, deliveries often occurred unanticipated leading to chaotic situations around the time of delivery.

A similar situation of limited monitoring of women’s condition was also seen after delivery. During observation of the period after delivery in the delivery room and on the ward, there were no assessments of the condition of women, such as checking of bleeding or uterine contraction.

Most respondents reported that they assessed progress of labour by conducting vaginal examinations and assessing cervical dilatation. Assessment of FHS was a rare response and was perceived as something to be done only when there was a delay in delivery. Similarly, blood pressure was reported as an examination to be conducted if the patient complained of headache or giddiness. Staff also reported being unable to monitor all women because of shortage of staff in the labour and maternity ward.

#### Some good practices followed

Some good practices were observed. These included staff encouraging women to mobilise and take food and tea during labour, staff allowing attendants to massage women’s backs, and women squatting before delivery. Some staff actively encouraged women to mobilise and eat while some showed passive support by not stopping them when attendants asked women to squat.

#### Harmful and unnecessary practices coupled with poor techniques

While monitoring was limited as described earlier, the staff also performed actions that are known to be potentially harmful and/or unnecessary. We present such practices we observed in Table 4 and the potential reasons for these practices from interviews in the following text.

**Table 4:** Description of some harmful and unnecessary practices observed in this study

Observation	Description
Fundal pressure	Routine at all facility levels, often given by ayah or sweeper, in the presence of nurses Much force used: standing on stool, by two people at a time
Perineum stretching rather than support	Perineal support was rarely provided Staff generally stretched the perineum, pulled the foetal head out
Frequent vaginal examinations	When multiple providers were present at a delivery, each conducted vaginal examinations despite seeing one done few minutes ago
Wrong interpretation of vaginal examination findings	Staff misdiagnosed the presenting part on vaginal examination
Improper management of third stage of labour	Uterotonic was not always administered/administered late after delivery of placenta Uterus was not stabilised when drawing placenta Placenta was never examined for completeness Manual exploration of uterus was routine
Episiotomy – timing and technique	When performed (though not routinely), episotomy was often performed too late Poor surgical technique – too small incisions, in small bits Local anaesthesia rarely given at the time of incision Observation: *The senior nurse came to the delivery room asking all staff ‘what’s the chaos here?’ She looked at the participant’s vulva to see the visible caput and said to the junior nurse ‘what’s this, what were you doing, why didn’t you call me?’ as she quickly put on sterile gloves. The head was at the perineum for some time and was delivered with episiotomy by the senior nurse. The newborn was bluish in colour and there was no cry at birth. (DH)*
Suturing	No local anaesthesia used during suturing No aseptic technique Unsterile suturing material used, often non-absorbable material used for skin suturing *Observation: The nurse started suturing with a small piece of catgut that was used up before she could suture the innermost layer. The sweeper gave her another small piece left over from an open packet (and unsterile), which the nurse refused and asked for a fresh one. The sweeper opened a packet of catgut and handed it to the nurse with naked hands. Whilst she was suturing the nurse accepted a phone call, adjusted her sari, and touched her spectacles with gloved hands. (CHC)*

Interviews with staff regarding the potentially harmful practices that were observed revealed a lack of appropriate knowledge and poor supervision. For instance, staff nurses felt fundal pressure was a useful way to deliver a baby stationed at the vulva and was helpful when they have no other support. They perceived it could potentially be harmful like causing a tear but also believed it expedites delivery and can thus potentially avoid stillbirth. Staff nurses reported that doctors did not discourage the use of fundal pressure, but often used this technique. Staff nurses said that they learned to manage deliveries using fundal pressure by observing other staff nurses during training.

The technique of stretching the perineum during second stage was believed to facilitate an easy delivery. Staff nurses stated that this technique could cause perineal abrasions and swelling, but this could be prevented by applying oil. Staff nurses appeared to be aware of the potential harms of frequent PV examinations but explained that attendants often demanded these examinations. They also reported that administration of antibiotics for all women was a good practice, as this prevented infections from multiple PV examinations.

#### Lack of readiness to provide routine care

The observations showed essential preparations to conduct a delivery were not in place. For instance, in most study facilities, the instruments tray was not ready. Rather the staff, in most cases the sweeper or the ayah, brought instruments, such as the artery forceps or scissors, when the delivery was imminent.

The ayah picked up the scissors left at the wash basin …. She dipped it in diluted Savlon, placed it in an instrument tray and gave it to the nurse. The nurse used the scissors to make an episiotomy. (DH)

Instruments used to conduct a delivery were not seen being sterilised or sent for sterilising to be ready for reuse. Instead, the instruments were left at the wash basin, and washed with tap water between deliveries. In the few facilities where an instrument tray was prepared, only one set of instruments was prepared, even though the staff often received more than one woman in labour at the same time.

The nurse prepared the delivery tray for the participant, to contain an artery forceps, scissors and thread, she picked these instruments with naked hands from a larger covered tray (not known if sterilised or not). When another woman arrived for delivery, she used these instruments. She then returned with the tray to the participant and cut the cord with the same gloves and using the same instruments she had just used. (PHC)

Preparing essential drugs for delivery was also seen to be poor in most facilities. For example, staff nurses in these facilities were unsure about whether routinely used drugs were available in the delivery room and so at the time of delivery, they asked attendants to buy drugs such as oxytocin or ergometrine from pharmacies outside the facilities.

The senior nurse asked the ayah to quickly pass the scissors and the junior nurse to load injection lignocaine. The junior nurse looked puzzled and replied ‘we don’t have it’. The ayah then told her it was on the trolley pointing to the lignocaine ampoule. (DH)

### Dominant staff, passive recipients of care

#### Staff provide some support during labour

Although there were many labouring women and relatively few staff in the DHs and CHCs, staff were sometimes seen providing support to the labouring women. For instance, a nurse at a CHC promptly counselled a participant who was crying when she delivered, as she had no attendant and felt lonely when she delivered. Staff were also seen to provide emotional support when a participant had heavy postpartum bleeding that required additional medication.

However, in most observations, women were verbally and physically abused, or ignored as described below.

#### Staff abuse and ignore women during delivery

Women were often abused by staff members during delivery, and this included slapping women and using foul language. The staff appeared to be anxious at the time of delivery and slapping the woman and forcing her to bear down faster was an attempt by the staff to accelerate the delivery. Generally, it was the sweepers or the ayahs who slapped women, while the nurses watched this happen without objection. The presence of attendants and the ASHA did not prevent such behaviour during delivery.

The sweeper hit the participant on her thighs asking her not to move. (CHC)

The ayah hit the participant on her hand and blamed her for taking out the IV needle (IV needle in the arm was dislodged while she was bearing down). (DH)

Staff appeared reluctant to quickly respond to women/attendants when they sought attention or there were danger signs. A woman admitted at a CHC felt diminished foetal movement, her mother later informed the observer that she had alerted the nurse but no one attended to her. On other occasions, attendants were seen to plead with the staff to examine or provide other care to their relative. However, staff often spoke disapprovingly of rural women and those with higher parity and seemed prejudiced against them. In addition, it was observed that when staff performed any examination or procedure, women were not informed of the findings.

A participant came to the delivery room; her loud scream drew everyone’s attention, except of the nurses in the delivery room. When requested by an attendant one nurse asked her to get the patient on the delivery table or make her sit down. When a senior nurse finally attended to her she found a breech presentation and imminent delivery. (DH).

#### Women are passive and accept dominance/disrespect

In addition to physical abuse, it was also observed that staff used coercion and dominated the situation. Physical force was used in the second stage of labour so that women adopted the desired position. Many times, the sweeper and/or ayah held women’s legs apart forcefully inhibiting any change in position. Despite obvious discomfort, neither women nor their attendants resisted. Staff threatened that if women did not comply, they would be asked to leave, or be left alone in the delivery room.

The participant was restless, bearing down with knees forced on to her chest. She grew more restless when fundal pressure was given and when staff hit her and talked in foul language. The attendants, the dai and few other women were around but none of them reacted to the staff hitting the participant who was screaming in pain. (CHC)

Neither participants nor their attendants resisted the abuse by the staff; they were tolerant and appeared helpless. At times when attendants noticed rude behaviour from the staff, they rather pleaded with them to take good care of the participant and forced the participant to cooperate with the staff.

When the nurse slapped the participant, the attendant woman looked sorry and helpless but stood silent looking at the floor. (CHC)

#### Attendants as ‘go-between’ patients and providers

We found that attendants had an essential role to play before and during delivery. Extending beyond merely providing companionship during delivery, they kept an eye on the labouring woman and sought medical attention when it was time for delivery. Using their own judgements in this way, attendants seemed to make up for the active monitoring of labour that was missing. Besides this, attendants were seen rushing to purchase and bring drugs and items like gloves around the time of delivery. Attendants supplied cloth pieces, which they brought from home, to the staff during delivery. These cloth pieces were a replacement for cotton, gauze, and towels expected to be routinely used during delivery. The attendants also received the newborn and implemented some aspects of newborn care like clothing the newborn and initiating feeding.

## Discussion

This article presents findings of an empirical assessment of quality of intrapartum care in the JSY programme. Although the success of the JSY at increasing coverage of facility births is recognised, delivering in an institution under the JSY did not ensure skilled birth attendance (SBA). Though staff followed some good practices, care was far from the requisite standards for SBA. These findings are important while exploring the possible reasons why the programme has not had an expected impact on maternal mortality decline in India. These findings highlight the need to ensure quality obstetric care and empower users to demand quality services while initiating programmes to increase demand for facility births.

This study investigated provision of care in the JSY in real time and in a comprehensive way by combining both clinical and social science perspectives. Using observation as a data collection tool allowed us to provide deeper insights into processes during intrapartum care that are difficult to assess after delivery. Although observation as a method has limitations, including subjectivity and limitations to feasibility, we have made the best effort at its use by following a scientific and systematic approach to produce credible findings. We have described factors that are likely to influence collection and interpretation of data namely the background of the observer and other researchers, the observer’s role and level of participation, and structure and timing of observation. During the data collection and analysis, we reflected on how our backgrounds and beliefs, such as being healthcare professionals, and women may influence what data were collected and how it has been interpreted. To reduce the influence of the observers’ presence on participant behaviour – the Hawthorne effect – visits were made to facilities before the observations were conducted, timing of facility visits was unannounced, and observations were one of many activities conducted during the 5-day facility visits. Although we cannot rule out the Hawthorne effect in this study, we do not think that it existed to a significant extent as staff did not ‘change’ negative practices, nor did they attempt to be more polite to women. We followed measures to increase trustworthiness and credibility such as using a pre-tested semi-structured guide and systematic piloting, selection of a range of representative facilities, random visits for observations and enrolling any consenting woman for the delivery observation, early transcription and confirmation of notes with a co-observer, triangulating delivery observations with subsequent staff interviews, and rigorous analysis by bringing multiple perspectives from co-authors.

In the following paragraphs, we discuss the key findings from this study focusing on their implications to quality of obstetric care in the context of a large cash transfer programme for facility births.

Our findings indicate that nurse-midwives have inadequate knowledge and skills to provide good quality care. This is evident from observations of limited monitoring during labour, poor techniques of providing care, routine use of harmful and unnecessary practices. The interviews supported these observations and identified possible underlying contributing factors such as inadequacies with pre-service and in-service training and the lack of supportive supervision. With the launch of the JSY, the Indian Government implemented a 3-week in-service skills building training programme for nurses – skilled birth attendant training. However, our findings of substandard practices despite training indicate the need to revisit the training content as well as the methodology. Our study of competence of staff in these settings has similar recommendations (12). It is important to note that studies have shown that in-service training on its own may not improve quality of care (13) but together with adequate support and supervision, it can improve quality of care (14).

Our findings indicate that despite some awareness of best practices, staff are unable to implement these practices in settings with human resource shortages and managerial problems and where quality is not an explicit priority. In their framework for SBA, Graham et al. (15) describe SBA as a result of skilled providers functioning in a setting conducive for appropriate care provision that they term as an ‘enabling environment’ for SBA. We found delivery rooms not being conducive to safe, women-friendly care, and poor coordination amongst staff as evident from gaps in the required infrastructure, material and human resources and importantly in the organisation of care and in the overall supervision and management of facilities. This can be seen in the way that non-qualified staff operate within the delivery room. The ayahs and sweepers appear to play an important role in the provision of care during delivery. However, some of this care is poor quality such as providing excessive fundal pressure, preparing instruments by swilling in water. It was clear from the observations that they are used to carrying out these activities, and this suggests that this type of care is accepted by all staff including managers. In the context of skilled staff shortages, the roles that unqualified staff can play in the delivery room is critical. However, they require adequate and relevant training and continuous supervision. Our study offers important insights showing how systems and structures fail to ensure an ‘enabling environment’ for quality care. The JSY programme in India was launched in the context of a health system reform that aimed to address the resource gaps and was, therefore, expected to provide the necessary enabling environment for the JSY to succeed. However, our findings show much remains to be done on this front. It is important to highlight that in addition to required physical and human resources, provision of quality care requires leadership and addressing challenges to professional accountability and governance.

Our findings provide evidence that women do not receive skilled attendance at birth during delivery in a facility under the JSY programme. An emphasis on coverage of facility births without ensuring quality of delivery care is likely to lead to a situation as in the Dominican Republic in 2000 (16), where maternal mortality remained high despite near-universal coverage of institutional births. Technically poor quality delivery care has been reported by several studies from other high maternal mortality contexts (17–19). Encouraging results on maternal mortality reduction have been shown in low- and middle-income countries such as Thailand, Malaysia, and Sri Lanka where they have focused not only on equitable coverage of delivery services but also on its effective coverage (20). Effective coverage of delivery care requires appropriate monitoring of labour and delivery to enable early recognition and appropriate treatment of obstetric complications. Our findings show inadequacies in such monitoring and hence the potential of high institutional birth coverage to translate into better health outcomes being unfulfilled. Hence, the question for the Indian context is how to use the opportunity to improve maternal and neonatal outcomes. The Indian situation is currently characterised by high maternal mortality, direct causes of maternal deaths predominate, access issues for some populations, but with a large shift to institutions for delivery care, characteristics that Souza et al. describe as those of countries in ‘third stage of obstetric transition’. They describe this stage as complex, during which a sharper decline in MMR occurs and facility care assumes a greater role in maternal mortality reduction (21). Improving quality of facility care, therefore, ought to be a priority action in India.

Although staff were empathetic in some situations, the findings of disrespect towards women reveal poor attitudes. Our findings of such behaviour by staff as routine indicate normalisation of disrespect and abuse during facility birth. In their framework portraying potential contributors to disrespect and abuse, Bowser and Hill (22) include such normalisation as one of the potential contributors. Other contributing factors in the framework resonate with the findings in our study. For example, the sufferers of such behaviour in our study are poor women who access care in public facilities and are not empowered to demand quality services, as might be their rich counterparts or male care seekers; poverty and female gender being contributors to abuse. Several factors at provider level included in Bowser’s framework were also found in our study, such as prejudice against rural and poor women of high parity, low staff morale and staff shortages as indicated during interviews with staff. In our study, all staff who provided disrespectful and abusive care were women. Many of these women are likely to experience low status and less respect, in a patriarchal society where gender-based violence is prevalent—in a nationwide survey 37.2% of married women reported experiencing violence (45.8% in MP) (23). This is an important consideration, as shown by Mumtaz et al. investigating why the SBA strategy in Pakistan was not as successful as anticipated. They reported an incongruity between the role of the midwife and the dominant social class and gender norms that devalue such a role (24). The facility environments in the settings observed lack standards for non-abusive care, supervision to maintain such standards and, importantly, any accountability mechanisms. These are also identified as contributors to disrespect and abuse (22).

Despite the abuse and disrespect, women and their attendants were quite uncritical, accepting the way care was provided. Our findings indicate a vicious cycle between women arriving very close to delivery and poor attention to women until the second stage of labour. In a recent study eliciting women’s response to abuse during facility birth in Tanzania, McMahon et al. (25) found responses ranging from acquiescent to assertive behaviours – with women preferring non-confrontational approaches and expressing sympathy for over worked staff. The authors provide a model for pathways from disrespectful care to dangerous delivery practices and argue that in response to abusive behaviour, clients might resort to minimising contact with health facilities by delivering at home, departing late for facilities, or leaving very early after delivery. Our findings of late arrival of women in labour could be a manifestation of women’s response to expected abusive behaviour as also shown by Hulton et al. (26).

Our findings indicate that although JSY has been successful at bringing women to institutions for delivery care, much remains to be done in terms of creating a demand for quality care. As service users, women can play important roles in improving the process of care ranging from participants of care, definers of quality, and reformers of care, and their participation is essential to bring professional accountability (27). Research suggests that addressing the invisibility, inferiority, and powerlessness of women accessing services could be potentially useful ways to improve maternal health services (28). It is recognised that such measures demand a systemic approach and are beyond the scope of a narrowly focussed cash transfer programme. Noteworthy evidence in this context is provided by the study by Barber and Gertler (29) who report better birth outcomes in Mexico’s Oppurtunidades programme are explained by better quality care. They further investigate the pathways by which this was achieved and report that Oppurtunidades affected quality through empowering women with knowledge about the programme content and skills and social support to negotiate better care.

Overall, our findings indicate a neglect of quality of care issue at different levels in the system, be it during training, service delivery, supervision, and monitoring. This suggests that a culture of quality has not developed in the health system. Disrespect and abuse during facility birth is identified as a barrier to improved quality of care (30). Recently, international efforts to influence policies to support respectful maternity care are increasing (31). Suggested measures to address include multilevel interventions – policy support, grievance redressal for women, community engagement, and provider training. Although the Indian government has instituted mechanisms that could enable women to register their grievances such as making complaints to facility superintendents, reports reveal low awareness about these mechanisms among women, lack of procedures for this and also that rural women refrain from complaining due to fear of reprisals (32).

With the launch of the JSY, the Ministry of Health also committed to set up quality improvement cells at facility and regional levels. Our findings of compromised quality question the effectiveness of these measures if implemented. Other measures known to improve the quality of maternal health services include criterion-based audits, near-miss audits, team work, and supportive supervision (33); however, concerns remain regarding the feasibility of implementing these interventions in resource-poor contexts that have poor record keeping. It is important to recognise that health worker performance is a complex behaviour and has multiple influences, and that multi-faceted interventions might be more effective at improving performance than any single-proven intervention (34). The answers to failures at delivering quality services may lie in embedding quality in the organisational cultures where poor quality is seen as an organisational weakness, and the emphasis is on improvement rather than on blaming individuals. Incorporating user, providers, and community perspectives in studies to further understand the factors underlying poor quality and inform quality improvement could be useful. Addressing both the clinical supervision needs of staff, especially those who function in isolation at PHCs, and overcrowding of DHs could be specific measures for consideration. Overall, our conclusions lead to recommendations in line with of those of Gaarder et al. (35) who reviewed evidence on impact of cash transfer programmes on health outcomes and highlight the need to find a balance between incentives, measures to improve quality of care, and empowering users. Further research could investigate how some facilities might provide good quality care in similar organisational environments. The possibilities of changing the way care is delivered and ensuring technical standards are maintained offer much hope for better gains from investments in the JSY cash transfer programme.

## Conclusions

In conclusion, our findings show unacceptably poor quality of care and a considerable gap between existing evidence-based recommendations (36) and delivery care practices despite births being conducted by trained professionals in public institutions. Although our study is limited by a small sample size and our conclusions are drawn from a qualitative paradigm, our findings bear relevance to other high maternal mortality provinces with increasing facility births in India and to other countries. Skilled attendance at birth being an accepted strategy for maternal mortality reduction, efforts now need to be focused on improving quality of care. Merely using proportion of births in facilities or attended by skilled providers is inadequate as a measure of progress in reaching maternal health goals and needs to be coupled with indicators reflecting quality of care received. While investing in demand side programmes like the JSY to increase coverage of skilled care at birth in low-income settings, it is a prerequisite to ensure the supply side of services delivers good quality care.
